# Heart failure in patients with coronary heart disease: Prevalence, characteristics and guideline implementation – Results from the German EuroAspire IV cohort

**DOI:** 10.1186/s12872-017-0543-0

**Published:** 2017-05-05

**Authors:** Caroline Morbach, Martin Wagner, Stefan Güntner, Carolin Malsch, Mehmet Oezkur, David Wood, Kornelia Kotseva, Rainer Leyh, Georg Ertl, Wolfgang Karmann, Peter U Heuschmann, Stefan Störk

**Affiliations:** 1 Comprehensive Heart Failure Center, University of Würzburg, Am Schwarzenberg 15, 97078 Wuerzburg, Germany; 2 Department of Medicine I, University Hospital of Würzburg, Wuerzburg, Germany; 3 Institute of Clinical Epidemiology and Biometry, University of Würzburg, Wuerzburg, Germany; 4 Department of Cardiovascular Surgery, University Hospital Würzburg, Wuerzburg, Germany; 5 Department of Cardiovascular Medicine, National Heart and Lung Institute, Imperial College London, London, UK; 6 Department of Public Health, University of Ghent, Ghent, Belgium; 7 Department of Medicine, Klinik Kitzinger Land, Kitzingen, Germany

**Keywords:** Heart failure, Coronary heart disease, Coronary artery disease, Guideline implementation, Guideline adherence, Awareness, EuroAspire, Mineralocorticoid antagonist, Pharmacotherapy, Preserved ejection fraction

## Abstract

**Background:**

Adherence to pharmacotherapeutic treatment guidelines in patients with heart failure (HF) is of major prognostic importance, but thorough implementation of guidelines in routine care remains insufficient. Our aim was to investigate prevalence and characteristics of HF in patients with coronary heart disease (CHD), and to assess the adherence to current HF guidelines in patients with HF stage C, thus identifying potential targets for the optimization of guideline implementation.

**Methods:**

Patients from the German sample of the *European Action on Secondary and Primary Prevention by Intervention to Reduce Events (EuroAspire) IV* survey with a hospitalization for CHD within the previous six to 36 months providing valid data on echocardiography as well as on signs and symptoms of HF were categorized into stages of HF: A, prevalence of risk factors for developing HF; B, asymptomatic but with structural heart disease; C, symptomatic HF. A Guideline Adherence Indicator (GAI-3) was calculated for patients with reduced (≤40%) left ventricular ejection fraction (HFrEF) as number of drugs taken per number of drugs indicated; beta-blockers, angiotensin converting enzyme inhibitors/angiotensin receptor blockers, and mineralocorticoid receptor antagonists (MRA) were considered.

**Results:**

509/536 patients entered analysis. HF stage A was prevalent in *n* = 20 (3.9%), stage B in *n* = 264 (51.9%), and stage C in *n* = 225 (44.2%) patients; 94/225 patients were diagnosed with HFrEF (42%). Stage C patients were older, had a longer duration of CHD, and a higher prevalence of arterial hypertension. Awareness of pre-diagnosed HF was low (19%). Overall GAI-3 of HFrEF patients was 96.4% with a trend towards lower GAI-3 in patients with lower LVEF due to less thorough MRA prescription.

**Conclusions:**

In our sample of CHD patients, prevalence of HF stage C was high and a sizable subgroup suffered from HFrEF. Overall, pharmacotherapy was fairly well implemented in HFrEF patients, although somewhat worse in patients with more reduced ejection fraction. Two major targets were identified possibly suited to further improve the implementation of HF guidelines: 1) increase patients´ awareness of diagnosis and importance of HF; and 2) disseminate knowledge about the importance of appropriately implementing the use of mineralocorticoid receptor antagonists.

**Trial registration:**

This is a cross-sectional analysis of a non-interventional study. Therefore, it was not registered as an interventional trial.

## Background

Chronic heart failure (HF) is a grave condition with high prevalence, increasing incidence and severely compromised prognosis [1], (https://www.destatis.de/DE/Publikationen/Thematisch/Gesundheit/Gesundheitszustand/GesundheitInDeutschlandPublikation.pdf). Clinical guidelines on diagnosis and treatment of acute and chronic HF [2] translate the complexity of scientific research findings into recommendations for daily practice. Adherence to current HF guidelines has been shown to substantially reduce disease severity and HF related symptoms as well as to improve prognosis regarding rehospitalization and mortality [3–13].

Despite their widespread recognition, it remains a notoriously difficult challenge to implement guidelines in clinical practice [14–19]. Deficits include omission of beneficial therapy, suboptimal patient outcome, and waste of resources [20, 21]. Identification of barriers for guideline implementation [16, 22] in programs or clinical trials showed improvements in managing chronic HF [9, 11, 13, 17, 23, 24]. By contrast, in routine patient care disease specific therapy remains frequently insufficient [5, 8, 10, 16–18, 25, 26].

One of the major diagnoses contributing to HF is coronary heart disease (CHD) [27]. The European Action on Secondary and Primary Prevention by Intervention to Reduce Events (EuroAspire) IV survey evaluated the quality of secondary CHD prevention according to the Joint European Societies guidelines in everyday clinical practice across Europe [28].

The aim of the present study was 1) to determine the prevalence and characteristics of HF in the German patients enrolled in EuroAspire IV and 2) to assess the implementation of ESC guidelines on HF [2] in this cohort of usually cared CHD patients to identify potential targets for optimization of guideline adherence.

## Methods

### Patient cohort

We present data from the German hospital arm of EuroAspire IV [28]. Patients between 18 and 79 years of age with CHD were invited to attend a study visit if they had been hospitalized due to a coronary event (coronary artery bypass grafting (CABG), percutaneous coronary intervention (PCI), acute myocardial infarction, or myocardial ischemia, the latter two receiving conservative therapy without intervention) six to 36 months prior to the anticipated study visit. The study was approved by the ethics committee of the Medical Faculty, University of Würzburg (Vote 58/12), and all patients provided written informed consent.

### Data collection

At the study visit (08/2012–03/2013), detailed information on medical history, medication, patient care, and behaviour was collected and physical examination (heart rate, weight, height, blood draw) was performed according to EuroAspire IV standards [28]. Information on the index event was collected by retrospective chart review [28]. Blood samples were processed, stored on-site, and analysed in batches. Selected parameters as lipid profile, glycosylated haemoglobin (HbA1c), and creatinine were analysed in the central EuroAspire IV Laboratory (Helsinki) [28].

In an additional heart failure module at the German study center, data on heart failure related symptoms were collected, and N-terminal pro-B-type natriuretic peptide (NT-proBNP) and high sensitive troponin T (hs-TnT) measured at the Central Laboratory of the University Hospital, Würzburg. During the face-to-face interview, patients were asked if they had ever been diagnosed with heart failure. The medical term (“Herzinsuffizienz”) and the German word (“Herzschwäche”) were explicitly mentioned and addressed by the interviewer. Angiography results of the index event were extracted from the hospital information system using the Comprehensive Heart Failure Center Data Warehouse [29]. When interpreting results from angiography reports, stenoses ≥50% were considered relevant.

### Echocardiography

Echocardiography scans were performed by one experienced physician sonographer (SG) using a Vivid Q® ultrasound scanner and a M4S transthoracic probe (GE Healthcare, Horten, Norway). The prespecified protocol adhered to recommendations formulated in current national and international guidelines [30–32]. All scans were stored digitally and analysed off-line (EchoPAC® PC Version 113; GE Healthcare, Buckinghamshire, Great Britain) by a European Association of Cardiovascular Imaging certified physician (CM) blinded to the patients´ history and study results. Left ventricular (LV) ejection fraction (EF) and LV end-diastolic volume were measured (Simpson’s biplane method, *n* = 428) or visually estimated. Regional wall motion abnormalities were assessed using a 16 segment model. Valve regurgitation was determined by the colour Doppler multiplane vena contracta method, and valve stenosis was quantified by continuous wave Doppler measurements [31, 33, 34]. LV mass index was calculated (cube formula) based on M-Mode derived measurements of interventricular septum and posterior wall [30].

### Categorization of heart failure

According to AHA/ACC guidelines [35], patients were grouped into stages of heart failure. To facilitate adequate categorization, the current guidelines emphasize the appropriate identification of “structural heart disease” [30, 36]. Accordingly, structural heart disease was diagnosed in the presence of at least one of the following: LV ejection fraction <50%, LV end-diastolic volume ≥ 97 mL/m^2^, LV mass index >95 g/m^2^ in females and >115 g/m^2^ in men, any stenosis of mitral or aortic valve, regurgitation > mild of mitral or aortic valve, left atrial area ≥ 30 cm^2^, septal e’ < 8 cm/s, or lateral e’ < 10 cm/s.

Heart failure related signs and symptoms included [37, 38] major criteria (paroxysmal nocturnal dyspnoea, orthopnoea, abnormal jugular venous distention, pulmonary rales, third heart sound) and minor criteria (ankle oedema, night cough, dyspnoea on exertion, hepatomegaly), while “symptomatic HF” was defined as 2 major or 1 major and 2 minor criteria being present concurrently [37, 38].

HF stage A was stated in the presence of risk factors for HF, but absence of both structural heart disease and HF related signs and symptoms. Stage B was defined as an asymptomatic patient at the study visit with documented structural heart disease. Stage C was assumed if a patient 1) already had or newly received the diagnosis of HF at discharge from index hospitalization; or was 2) symptomatic and had structural heart disease at the study visit. Stage C patients who had documented systolic dysfunction (LVEF ≤40%) at the index event and/or at the study visit were further subclassified into HF with reduced LVEF (HFrEF) in whom HF guidelines regarding pharmacotherapy could be applied.

### Cardiovascular risk factors and comorbidities

Hypertension was defined as suggested by the German Society of Cardiology [35] as systolic blood pressure ≥ 140 mmHg or diastolic blood pressure ≥ 90 mmHg. Cut-off values differed for older or diabetic patients and those with chronic kidney disease [28].

### Implementation of guideline recommendations on pharmacotherapy

Quality of guideline implementation in stage C patients was evaluated using the 2008 European Society of Cardiology Guidelines for the diagnosis and treatment of acute and chronic HF applicable at the time of the study visit [2]. The Guideline Adherence Indicator (GAI-3) was calculated (range 0%–100%) for each patient as number of drugs taken divided by number of drugs indicated considering indication and contraindication of beta-blockers, angiotensin converting enzyme inhibitors/angiotensin receptor blockers (ACEi/ARB), and mineralocorticoid receptor antagonists (MRA) [5, 7, 8, 12]: a) ACEi/ARB if LVEF ≤40% and New York Heart Association functional class (NYHA) I-IV, b) beta-blocker if LVEF ≤40% and NYHA II-IV or NYHA I and prior myocardial infarction, and c) MRA if LVEF ≤35% and NYHA III-IV but absence of major kidney dysfunction.

### Adherence to non-pharmacological advice

In a structured interview, participants were asked if they had ever been given advice by a doctor or other health care professional regarding an increase in exercise, smoking cessation, or change of diet (sodium restriction, fat reduction, calory restriction, eating more fish), and if they had followed such advice. Adherence was assessed as number of patients who followed a given advice divided by the number of patients who had been given the respective advice.

### Data analysis

Statistical analysis was performed using SPSS (Version 23, SPSS Inc., Chicago, USA). Frequencies are reported as n (%) and quantitative data expressed as mean (standard deviation) or median (quartiles) depending on normality of distribution. We employed Q-Q plots to graphically check the normality assumption of individual variables. Univariable analyses across categories (e.g. HF stages, study visit vs. hospital discharge, LVEF groups) were performed using Fisher’s exact, ANCOVA or Kruskal-Wallis test, as appropriate. Two-sided *p*-values <0.05 were considered statistically significant. *P*-values were not corrected for multiple testing as the study was exploratory.

## Results

Of the 536 patients enrolled at the German study center in 2012/13, 509 subjects had valid information on medical history, HF symptoms and echocardiographic results, and could be classified into stages of HF (Table 1): stage A, *n* = 20 (3.9%); stage B, *n* = 264 (51.9%); stage C, *n* = 225 (44.2%); 94 patients were diagnosed with HFrEF. Patients in higher HF stages were older (56 years in stage A, 67 years in stage B, 68 years in stage C), had a longer duration or CHD (2.0 years, 2.8 years, 2.9 years, respectively), were more likely to be hypertensive (15%, 49%, 42%, respectively), and suffered more often from chronic kidney disease (5%, 19%, 35%, respectively; all p for trend ≤0.007). Time since index event, the treatment (CABG vs. PCI/stent, conservative) and urgency (acute vs. elective) of the index event and the number and localization of affected coronary arteries were not significantly associated with HF stages. Patients in HF stage B and C also had significantly higher levels of NT-proBNP (76 pg/ml, 146 pg/ml, 245 pg/ml) and hs-TnT (6.1 pg/ml, 8.8 pg/ml, 10.0 pg/ml; p for trend ≤0.012).

**Table 1:** Characteristics, cardiovascular risk factors, comorbidities and measurements according to heart failure stages

	Stage of Heart Failure			
	A	B	C	p
N (%)	20 (3.9)	264 (51.9)	225 (44.2)	
Age (years), mean (SD)	56.3 (8.3)	66.8 (8.2)	68.1 (9.1)	<0.001^1^
Male sex, n (%)	16 (80.0)	221 (83.7)	180 (80.0)	0.514^2^
Time since index event (months), mean (SD)	20.4 (8.6)	20.8 (9.1)	22.2 (8.7)	0.194^1^
Index event				
Acute, n (%)	20 (100)	259 (98.5)	223 (99.6)	0.949^2^
Primary CHD event, n (%)	4 (20.0)	71 (26.9)	54 (24.0)	0.696^2^
Affected vessels				
Main left coronary artery, n (%)	0	15 (6.8)	18 (9.5)	0.364^2^
LAD, n (%)	11 (64.7)	145 (65.9)	140 (74.1)	0.171^2^
RCX, n (%)	9 (52.9)	106 (48.2)	93 (49.2)	0.912^2^
RCA, n (%)	7 (41.2)	117 (53.2)	96 (50.8)	0.62^2^
Number of affected vessels				0.699^2^
1, n (%)	9 (52.9)	100 (45.5)	85 (45.0)	
2, n (%)	6 (35.3)	68 (30.9)	62 (32.8)	
3, n (%)	2 (11.8)	44 (20.0)	40 (21.2)	
Duration of CHD (years), median (IQR)	2.0 (1.4–3.4)	2.8 (1.9–10.1)	2.9 (2.0–9.9)	0.027^3^
Treatment for index event				0.417^2^
Conservative, n (%)	6 (30.0)	38 (14.4)	37 (16.4)	
CABG, n (%)	2 (10.0)	45 (17.0)	32 (14.2)	
PCI, n (%)	12 (60.0)	181 (68.6)	156 (69.3)	
Cardiovascular risk factors and comorbidities				
Smoking^a^, n (%)	5 (25.0)	27 (10.2)	19 (8.4)	0.071^2^
Obesity^b^, n (%)	8 (40.0)	96 (36.5)	80 (35.9)	0.932^2^
LDL > = 2.6 mmol/L, n (%)	8 (44.4)	123 (49.0)	88 (41.9)	0.324^2^
Hypertension^c^, n (%)	3 (15.0)	128 (48.7)	94 (41.8)	0.007^2^
Diabetes melltius^d^, n (%)	7 (35.0)	102 (38.8)	87 (39.4)	0.947^2^
Chronic kidney disease^e^, n (%)	1 (5.0)	51 (19.3)	76 (34.7)	<0.001^2^
Measurements				
BMI (kg/m2), median (IQR)	27.7 (25.1–33.5)	28.4 (26.3–31.1)	28.7 (26.1–31.1)	0.926^3^
Systolic BP (mmHg), median (IQR)	119 (114–128)	138 (126–152)	133 (123–148)	<0.001^3^
Diastolic BP (mmHg), median (IQR)	75 (66–80)	81 (73–88)	80 (73–86)	0.017^3^
Heart rate (min-1), median (IQR)	57 (55–64)	61 (56–69)	63 (58–70)	0.015^3^
Sinus rhythm, n (%)	20 (100)	238 (93.0)	186 (90.7)	0.368^2^
LDL Cholesterol (mmol/L), median (IQR)	2.6 (2.2–3.4)	2.6 (2.1–3.1)	2.5 (2.0–3.1)	0.623^3^
HbA1c (%), median (IQR)	5.6 (5.3–6.0)	5.7 (5.5–6.3)	5.8 (5.4–6.2)	0.376^3^
NT-proBNP (pg/mL), median (IQR)	76 (35–146)	146 (78.5–312.5)	245 (113.3–626.8)	<0.001^3^
Hs-TnT (pg/mL), median (IQR)	6.1 (5.4–18.5)	8.8 (6.6–11.8)	10.0 (7.1–14.9)	0.012^3^

*SD* standard deviation, *LAD* left anterior descending coronary artery, *RCX* left circumflex coronary artery, *RCA* right coronary artery, *CHD* coronary heart disease, *IQR* interquartile range, *CABG* coronary artery bypass graft, *PCI* percutaneous coronary intervention, *LDL* low densitiy lipoprotein, *BMI* body mass index, *HbA1c* haemoglobin A1c, *NT-proBNP* N-terminal pro B-type natriuretic peptide, *hs-TnT* high-sensitive troponin T, *BP* blood pressure

1 ANCOVA

2 Fisher’s exact test

3 Kruskal-Wallis test

^a^current smoker

^b^BMI ≥ 30 kg/m^2^

^c^systolic blood pressure ≥ 140 mmHg or diastolic blood pressure ≥ 90 mmHg or in patients with diabetes: systolic ≥140, diastolic ≥85 mmHg; >80 yrs.: systolic ≥150, diastolic ≥90 mmHg, chronic kidney disease systolic ≥130, diastolic ≥90 mmHg

^d^self reported or impaired fasting glucose or impaired glucose tolerance

^e^glomerular filtration rate < 60 mL/min/kg

### Echocardiography

In advanced HF stages, patients had significantly lower LVEF, larger LV and left atrial volumes, higher LV mass, higher prevalence of regional LV wall motion abnormalities, and higher ratios of mitral valve inflow velocity (E) over average diastolic mitral ring velocity (e´) as measure for diastolic dysfunction (Table 2).

**Table 2:** Echocardiographic measurements in all patients and according to heart failure stages

		Heart Failure Stage			
	Total	A	B	C	p
N (%)	509	20 (3.9)	264 (51.9)	225 (44.2)	
LVEF (%), median (IQR)	58 (53–62)	64 (58–67)	59 (55–63)	56 (50–61)	<0.001^1^
LVEF categories, n (%)					
< 30%	7 (1.4)	0	2 (0.8)	5 (2.3)	0.041^2^
30–39%	23 (4.5)	0	10 (3.8)	13 (6.1)	
40–52%	80 (15.6)	1 (5.0)	32 (12.1)	43 (20.1)	
> 52%	402 (78.5)	19 (95.0)	220 (83.3)	153 (71.5)	
Wall motion abnormalities, n (%)	138 (28.1)	2 (10.0)	59 (23.4)	74 (35.7)	0.003^2^
LVEDVI (ml/m^2^), median (IQR)	60 (50–72)	49 (40–56)	60 (50–69)	61 (53–79)	<0.001^1^
LV mass index (g/m^2^), median (IQR)	93 (78–110)	82 (64–95)	92 (77–108)	99 (82–120)	<0.001^1^
LAA (cm^2^), median (IQR)	20 (17–23)	18 (16–20)	20 (17–23)	20 (17–24)	0.038^1^
E/e´, median (IQR)	9 (7.2–11.4)	6.8 (5.5–8.4)	8.8 (7.2–10.8)	9.5 (7.5–12.1)	<0.001^1^

*LV* left ventricle, *EF* ejection fraction, *IQR* interquartile range, *LVEDVI* LV end-diastolic volume index, *LAA* left atrial area, *E/e´* LV filling index (transmitral inflow velocity to annular velocity ratio)

^1^Kruskal-Wallis test

^2^Fisher’s exact test

### Patient care

In HF stages A, B, and C, the main caregivers in regards to CHD within the previous 3 months were primary care physicians for *n* = 3 (15.5%), *n* = 70 (26.6%), and *n* = 48 (21.9%) patients, and cardiologists for *n* = 15 (75%), *n* = 160 (60.8%), and *n* = 160 (71.7%) patients, respectively. By contrast, *n* = 2 (10.0%), *n* = 17 (6.5%), and *n* = 7 (3.1%) patients in stages A, B, and C reported on no main caregiver for CHD at all. While HF was documented at index hospitalization in 202 (89.8%) stage C patients, at the study visit only 39 (19%) of these subjects and 44 (19.7%) stage C patients in total, reported to be aware of this diagnosis at the study visit interview.

### Guideline-adherence

In 214 out of 225 (96%) patients with HF stage C, current pharmacotherapy could be assessed. From discharge after index hospitalization to the study visit (Table 3), there was a significant reduction in prescription frequency of MRA (13% vs. 8.4%) and beta-blocker (93% vs. 85%). A contraindication was prevalent at the time of the study visit in 7 of 27 (26%) patients stopping beta-blocker, and in one of 18 (6%) patients stopping MRA therapy. NYHA class was not significantly different between LVEF categories, but patients with lower LVEF had significantly higher hs-TnT and NT-proBNP values. Prescription rates of beta-blocker and ACEi/ARB did not differ significantly, but patients with reduced LVEF were more often on MRA and diuretic medication (Table 4). In patients with HFrEF, quality of pharmacotherapy recommended by European guidelines applicable at the time of the study visit [2] varied markedly across substance classes: current pharmacotherapy fulfilled guideline recommendations [2] regarding ACEi/ARB in 86 (91.5%), regarding beta-blocker in 85 (90.4%), and regarding MRA in 4 (57%) HFrEF patients with the respective indication. Total GAI-3 was 96.4% (*n* = 87 with valid information on indication and contraindications of all three substance classes). No apparent difference in the quality of HF pharmacotherapy according to GAI-3 was detected between subjects treated predominantly by cardiologists vs general practitioners (*p* = 0.88).

**Table 3:** Pharmacotherapy of patients in HF stage C at discharge and at the study visit

	Discharge	Study visit	p
Beta blocker, n (%)	210 (93.3)	191 (84.9)	0.002^1^
ACEi/ARB, n (%)	197 (87.6)	187 (83.1)	0.154^1^
MRA, n (%)	30 (13.3)	19 (8.4)	0.043^1^
Loop diuretic, n (%)	66 (29.3)	72 (32.0)	0.451^1^
Thiazide diuretic, n (%)	35 (15.6)	42 (18.7)	0.382^1^
Glycoside, n (%)	12 (5.3)	14 (6.2)	0.625^1^

*N* = 225, age 68.1 (9.1) years, males 180 (80%)

*ACEi/ARB* angiotensin-converting-enzyme inhibitor/angiotensin-receptor blocker, *MRA* mineralocorticoidreceptor antagonist

^1^Fisher’s exact test

**Table 4:** Characteristics, measurements and pharmacotherapy of patients in HF stage C at the study visit according to left ventricular ejection fraction

Left ventricular ejection fraction	<40%	40–52%	>52%	p
N (%)	18 (8.4)	43 (20.1)	153 (71.5)	
Age year, mean (SD)	69 (8.6)	69 (9.3)	67 (9.2)	0.409^1^
Males, n (%)	17 (94.4)	36 (83.7)	117 (76.5)	0.16^2^
NYHA class, n (%)				0.104^2^
I	5 (29.4)	9 (21.4)	53 (35.1)	
II	5 (29.4)	20 (47.6)	66 (43.7)	
III	5 (29.4)	12 (28.6)	30 (19.9)	
IV	2 (11.8)	1 (2.4)	2 (1.3)	
Hs-TnT, median (IQR)	14.3 (8.6–22.9)	12.3 (8.1–17.8)	8.9 (6.7–13.0)	0.001^3^
NT-proBNP, median (IQR)	783 (371.5–1960.5)	495 (255.5–1061.8)	189 (86.5–401.0)	<0.001^3^
Beta blocker, n (%)	18 (100)	38 (88.4)	125 (81.7)	0.085^2^
ACEi/ARB, n (%)	16 (88.9)	36 (83.7)	125 (81.7)	0.872^2^
MRA, n (%)	3 (16.7)	7 (16.3)	7 (4.6)	0.011^2^
Loop diuretic, n (%)	12 (66.7)	21 (48.8)	35 (22.9)	<0.001^2^
Thiazide diuretic, n (%)	2 (11.1)	1 (2.3)	37 (24.2)	0.001^2^
Glycoside, n (%)	3 (16.7)	4 (9.3)	7 (4.6)	0.074^2^

*N* = 225, age 68.1 (9.1) years, males 180 (80%)

*NYHA* New York Heart Association functional class, *hsTNT* high sensitive troponin T, *NT-proBNP* N-terminal pro B-type natriuretic peptide, *ACEi/ARB* angiotensin-converting-enzyme inhibitor/angiotensin-receptor blocker, *MRA* mineralocorticoid receptor antagonist, *GAI-3* Guideline Adherence Indicator: number of drugs indicated divided by number of drugs prescribed according to 2008 ESC HF guideline [2]

^1^ANCOVA

^2^Fisher’s exact test

^3^Kruskal-Wallis test

One hundred and eighty-three (81%) stage C patients were on statin therapy. Of those, in 66 and 160 (36% and 87%) LDL levels were above 2.6 mmol/l and 1.8 mmol/l, respectively. Non-pharmacological advice regarding physical exercise, smoking cessation, sodium restricted diet, fat reduced diet, calory reduced diet, and a diet containing more fish was followed in 103 (70.1%), 13 (68.7%), 86 (81.9%), 115 (82.7%), 62 (66.7%), and 92 (79.3%) of respectively adviced stage C patients. Patients, who were aware of having heart failure, followed non-pharmacological advice more often compared to unaware patients (exercise 72.4% vs. 69.0%; smoking 100% vs. 81.8%; sodium restricted diet 79.2% vs. 82.5%; fat reduced diet 82.2% vs. 82.4%; calory reduced diet 90.5% vs. 58.6%; diet containing more fish 77.8% vs. 79.8%).

## Discussion

### Prevalence and characteristics of HF in CHD

In this German sample of the EuroAspire IV cohort of patients hospitalized for CHD within the previous six to 36 months, we found a high prevalence of HF stages B and C. Patients in HF stage C were older, had a longer duration of CHD and were more likely to suffer from hypertension and chronic kidney disease. We could not observe associations between HF stage and the urgency or therapy of the index CHD event that classified for participation in EuroAspire IV, or the affected coronary vessels. Although HF stage C patients exhibited more regional wall motion abnormalities, a majority of stage C patients presented with preserved LVEF and the median LVEF was still in the normal range.

It is reassuring, that only a minority of patients with a coronary event requiring hospital admission had HF with reduced ejection fraction. This, at least in parts, might be due to the optimization of emergency pre-hospital and in-hospital care and improved treatment options preventing extensive myocardial damage [39]. On the other hand this highlights the importance of arterial hypertension in patients with CHD, which was the most important risk factor for HF stage C in our sample (along with chronic kidney disease, which again is predominantly caused by arterial hypertension [40]). Hypertension is an established independent risk factor for CHD in all populations accounting for approximately 47% of ischemic events [40, 41]. It further represents a major component in the progression of left ventricular dysfunction into symptomatic HF [40]. With increased LV mass, enlarged left atrium, diastolic dysfunction and preserved LVEF, a substantial part of our stage C patients showed the typical echocardiographic signs of hypertensive heart disease indicating a co-existence of both, CHD and hypertensive heart disease. Physicians, caring for CHD patients, should be aware of the high likelihood of HF with preserved LVEF in those patients. Further prospective studies are needed to investigate the distinct pathogenesis of asymptomatic myocardial dysfunction aggravating into symptomatic HF [42] and to clarify the importance of aggressive blood pressure control in this special patient collective.

### Guideline adherence in stable chronic CHD patients

Although the diagnosis of HF was clearly stated in the discharge letter of the index hospital stay in the majority of stage C patients, only about one fifth of these patients were aware of this diagnosis at the study visit interview. The majority of stage C patients exhibited preserved LVEF at the study visit; in these patients evidence to support HF treatment is limited. However, a substantial number of patients had had impaired LVEF at the index event or had reduced LVEF at the time of the study visit thus meeting the criteria for the diagnosis of HFrEF and qualifying to apply guidelines for pharmacotherapy. HF related pharmacotherapy followed guideline recommendations in a substantial part of HFrEF patients. There was a trend towards lower GAI-3 in patients with lower LVEF, mainly due to lower rates of MRA prescription. Compared to discharge pharmacotherapy, significant changes in medication had occurred at the study visit with less frequent prescription of beta-blocker and MRA, which could only in part be explained by contraindications prevalent at the study visit. There was no association between GAI-3 at study visit and main caregiver within the previous 3 months.

Guideline adherence in treatment of HF has been shown to substantially improve patients´ prognosis regarding morbidity, rehospitalization, and mortality [3–7, 9, 12, 13]. The MAHLER study defined a guideline adherence indicator (GAI-3) concerning the three prognostic relevant substances beta-blockers, ACEi/ARB, and MRA [7]. GAI-3 was highly predictive of favourable changes of LVEF and LV end-diastolic diameter [9], frequency of and time to hospitalization for HF [7], and survival [5, 9, 10, 12].

Although an improved guideline adherence over the past decades has been suggested (https://www.destatis.de/DE/Publikationen/Thematisch/Gesundheit/Gesundheitszustand/GesundheitInDeutschlandPublikation.pdf), [4, 13, 23], guideline implementation and long-term adherence remain challenging (https://www.destatis.de/DE/Publikationen/Thematisch/Gesundheit/Gesundheitszustand/GesundheitInDeutschlandPublikation.pdf), [15, 18, 25]. Recent data from 15 randomly chosen general practitioners in the metropolitan region of Hamburg, Germany, showed high prescription rate of ACEi/ARB (76%), beta-blockers (73%), and MRA (18%) in outpatients with chronic HF [43]. However, after collective review of patient records and subsequent counselling, prescription rates could further be improved (ACEi/ARB: 87%, beta-blocker: 84%), and GAI-3 increased from 22% to 56% [43].

In our study sample, we found comparable prescription rates of MRA but higher rates for beta-blockers and ACEi/ARB. This might, at least in parts, be due to the fact, that all patients had a hospital stay within the previous 3 years and had been discharged with optimized pharmacotherapy. Further, beta-blockers as ACEi/ARB also constitute corner stones in the treatment of both CHD and hypertension. Nevertheless, we observed that MRA were prescribed at lower than desired frequency, in particular regarding the time interval from hospital discharge to the study visit, and lower GAI-3 in patients with more reduced LVEF at the time of the study visit due to lower prescription rates of MRA. Since this observation is based on small numbers, it awaits corroboration in larger samples. The hesitation to prescribe MRA might be due to the higher likelihood of potential adverse events requiring a closer patient monitoring, but also because the importance of MRA regarding favourable prognosis may have not yet sufficiently transferred to the outpatient setting [43].

Non-pharmacological advice [2] regarding physical exercise, smoking cessation, and a healthy diet was followed in a substantial part of stage C patients. Further, as part of secondary prevention for CHD, four fifth of stage C patients were on statin therapy and CHD treatment goals were reached in about two thirds of these patients. Although lipid lowering therapy is a corner stone in the secondary prevention of CHD, it should be carefully reviewed in patients with symptomatic HF as there is no evidence for statins improving the prognosis of patients with symptomatic HF [2], and lower total cholesterol has been shown to be associated with increased mortality in HF patients [44].

Overall, our numbers suggest successful implementation of HF guideline recommendations in usual patient care, similarly for general practitioners and cardiologists, although there is still room for improvement.

Deficient patients´ awareness of their disease is one of the major problems in the implementation of guidelines. The low awareness of previously documented HF, assessed in the context of an interview, in our study sample is in line with the findings of a contemporary, multinational convenience sample of subjects attending the HF Awareness Day Initiatives in 2013 revealing low awareness of HF and its typical signs and symptoms in the lay public as well as important misconceptions regarding the importance and the prognostic implications of HF [45]. The German National Disease Management Guideline “Chronic Heart Failure” [27] as well as the Guideline on HF of the German Society of Primary Care [46] highlight the importance of information about and acceptance of the own disease for therapy adherence. Therefore, programs to increase knowledge about and how to live with the disease as well as potential involvement of family members into patient care are essential [27, 46].

Our study showed a fairly high, but still not sufficient level of implementation and subsequent adherence to current HF guidelines in patients with CHD and chronic HF. These results suggest that increasing patients´ awareness of their diagnosis and importance of HF as well as spreading the knowledge about the importance of MRA in the pharmacotherapy of chronic HF may be promising targets for the improvement of guideline implementation.

### Limitations

Our sample might represent a best practice scenario due to its selection, but our data still indicate that efforts into the direction of guideline implementation are feasible. Selection might have occurred in favour of more compliant patients attending the study [28], but our study participation selection process did not take influence on the main caregiver. Further, there were a few patients with an LVEF <30% but without signs and symptoms of HF. Although signs of HF were assessed very carefully at the study visit, HF related symptoms could only be assessed by self-reporting, i.e. no technical method was used (such as spiroergometry) to objectify dyspnoea on exertion. Nevertheless, assessment of the Framingham criteria is a common and well evaluated method to assess HF and has shown high diagnostic and prognostic accuracy [37, 38]. Regarding guideline-adherence, only contraindications prevalent at the time of the study visit could be taken into account. Further, the total number of aware patients was too small to reliably investigate the association between awareness and non-adherence to guidelines.

## Conclusions

In the German EuroAspire IV sample of CHD patients, we found a high prevalence of both asymptomatic HF stage B and overt HF stage C. A majority of stage C patients had a preserved ejection fraction and revealed hypertension and chronic kidney disease as major HF-related factors. Although pharmacotherapy of HFrEF patients fairly well complied with current HF guidelines in patients with higher LVEF, its implementation was worse in patients with more reduced LVEF. We identified two major targets to optimize guideline implementation: 1) to increase the patients´ awareness of the diagnosis and importance of HF and 2) to spread the knowledge about the prognostic impact of MRA in the pharmacotherapy of chronic HF.

## Abbreviations

ACC: American College of Cardiology
ACEi: Angiotensin converting enzyme inhibitor
AHA: American Heart Association
ARB: Angiotensin receptor blocker
CABG: Coronary artery bypass grafting
ESC: European Society of Cardiology
EuroAspire: European Action on Secondary and Primary Prevention by Intervention to Reduce Events survey
CHD: Coronary heart disease
GAI: Guideline adherence indicator
HbA1c: Glycosylated haemoglobin A1c
HF: Heart failure
hsTNT: High sensitive troponin T
LV: Left ventricle
LVEF: Left ventricular ejection fraction
MRA: Mineralocorticoid receptor antagonist
NT-proBNP: N-terminal pro B-type natriuretic peptide
NYHA: New York Heart Association functional class
PCI: Percutaneous coronary intervention
